# Pro-death activity of a BH3 domain in an aquaporin from the protozoan parasite *Leishmania*

**DOI:** 10.1038/cddis.2016.229

**Published:** 2016-07-28

**Authors:** Carlos Mario Genes, Héctor de Lucio, Pedro Alejandro Sánchez-Murcia, Federico Gago, Antonio Jiménez-Ruiz

**Affiliations:** 1 Departamento de Biología de Sistemas, Universidad de Alcalá, Alcalá de Henares, 28805 Spain; 2 Departamento de Ciencias Biomédicas, Universidad de Alcalá, Alcalá de Henares, 28805 Spain

The decision of a given cell to undergo cell death is determined, in most cases, by the interplay among pro- and anti-apoptotic members of the Bcl-2 family of proteins. Anti-apoptotic proteins such as Bcl-2, Bcl-X_L_, and MCL-1 maintain cell survival by binding and sequestering their pro-apoptotic counterparts, which can be classified as Bcl-2 homology 3 (BH3)-only or (BH1–BH3) multi-domain proteins.

BH3-only proteins act as sentinels that can be divided into two different groups: (i) activator BH3s which, upon specific upstream signals, directly activate BAX or BAK and (ii) sensitizer BH3s that bind to the anti-apoptotic family members thereby blocking their association with BAX or BAK. In all cases, the activity of BH3-only proteins relies on the binding of their BH3 domains into a deep elongated cleft present in the multi-domain pro- or anti-apoptotic members.^1^ Interactions between BH3-only proteins and the anti-apoptotic members of the family can be readily detected by common methods such as co-immunoprecipitation or two-hybrid interactions in yeasts. In contrast, their binding to the pro-apoptotic BAX or BAK is harder to detect because the much weaker interactions involved are only observed in the presence of non-ionic detergents.^2^

BH3 domains consist of an amphipathic α-helix with residues that broadly comply with the Prosite PS01259 pattern ([LIVAT]-x(3)-L-[KARQ]-x-[IVAL]-G-D-[DESG]-[LIMFV]-[DENSHQ]-[LVSHRQ]-[NSR]). Replacement of some key residues from this motif signature abrogates the pro-apoptotic function of the proteins. Thus, the BH3 domain of the BH3-only proteins is both necessary and sufficient for their apoptotic function.

Apoptotic cell death has been deeply studied in metazoans for more than two decades but the presence of apoptosis-like processes in protozoans is still a matter of debate. Programmed cell death (PCD) in these single-celled organisms might constitute an efficient strategy to promote the reproductive success of some members of a clonal population, but the almost absence of demonstrated molecular pathways that regulate this process is still a major concern that hampers acceptance of the existence of PCD in protozoa. Endonucleases G^3, 4, 5^ and metacaspases^6, 7, 8^ constitute the only two relevant enzymes in single-celled organisms for which a role in apoptosis-like processes has been demonstrated so far. Regarding the Bcl-2 family of proteins, it has been shown that ectopic expression of some of their members can inhibit (e.g. Bcl-X_L_) or promote (e.g. Bax) cell death processes when expressed in yeast^9, 10^ or *Leishmania* cells.^11, 12^ However, up till now the yeast BH3-only Ybh3p is the unique protein from a single-celled organism that contains a functional BH3 domain with a demonstrated pro-death activity.^13^

In our recent *Cell Death Discovery* article (doi:10.1038/cddiscovery.2016.43),^14^ we show that a previously described aquaporin, now renamed Li-BH3AQP, contains an amino acid stretch at its C-terminus (283-MYLALQNLGDEV-294) that closely matches the consensus PS01259 motif signature and is responsible for the pro-death behavior of this protein in response to drug treatment. The domain is completely conserved in orthologs from *L. donovani*, *L. major*, *L. mexicana*, *L. braziliensis*, and *L. panamensis*.

Molecular modeling work based on the crystal structures of aquaporins Aqy1 from *Pichia pastoris* and AqpM from *Methanothermobacter marburgensis* revealed that each monomer in Li-BH3AQP is likely to comprise six full membrane-spanning helices plus two intracellular and three extracellular loops. In addition, each monomer presents two half membrane-spanning helices, which make up a seventh pseudo-transmembrane segment that contains one Asn–Pro–Ala aquaporin signature motif located near the center of the water channel. The putative BH3 domain is thus projected out toward the opposite face of the channel where it could be accessible to Bcl-2-like proteins (Figure 1). Regarding the quaternary structure of the protein, Li-BH3AQP would be organized as a homotetramer within the lipid membrane, with both N- and C-termini oriented toward the cytosol. Our molecular model of the putative Bcl-X_L_:Li-BH3AQP complex shows the hydrophobic side chains of Met283, Leu287, Leu290, and Val294 in the 283-MYLALQNLGDEV-294 stretch buried inside four well-defined pockets in Bcl-X_L_ that were structurally characterized previously in the complex between this anti-apoptotic protein and Bim.^15^ The negatively charged carboxylate of Asp292 in Li-BH3AQP is also shown to occupy an equivalent position to that of Asp99 in Bim.

Co-immunoprecipitation of a Myc-tagged version of Li-BH3AQP and a flag-tagged version of human Bcl-X_L_ in HEK293T cells gave us the first indication that these two proteins are able to interact in a cellular context. This interaction was subsequently demonstrated to occur also in a yeast two-hybrid system using Bcl-X_L_ (fused to the DNA-binding domain of GAL4) as the 'bait' and Li-BH3AQP (fused to the activation domain of GAL4) as the 'prey'.

In addition to its ability to bind to Bcl-X_L_, Li-BH3AQP truly behaves as a pro-apoptotic molecule, as indicated by the significant reduction in the number of viable cells observed after its ectopic expression in HeLa cells. As expected, simultaneous overexpression of Bcl-X_L_ restored cell viability.

Regarding the role of the protein in *Leishmania* parasites, Li-BH3AQP overexpression caused a significant reduction in the number of live promastigotes after treatment with staurosporine or antimycin A. Overexpression of a Li-BH3AQP version in which two key residues from the BH3 domain were replaced by lysine (Li-BH3AQP^L287K/D292K^) did not increase parasite sensitivity to these two drugs. We take these findings as a demonstration of the implication of BH3 domain in the pro-death role of this protein. In all cases, increases in cell death could be correlated with changes in characteristic markers of apoptosis-like death, such as chromosomal DNA degradation. As shown for many other death-regulating proteins, Li-BH3AQP does also display a pro-survival role that could be detected when parasites overexpressing it were exposed to environmental conditions in which solute trafficking through the membranes is especially relevant. Thus, cell death caused by nutrient deprivation or hypotonic stress was significantly reduced when Li-BH3AQP was overexpressed and this pro-life role was maintained in the Li-BH3AQP^L287K/D292K^ version of the protein.

Taken together, the results presented in our *Cell Death Discovery* article (doi:10.1038/cddiscovery.2016.43) support the inclusion of Li-BH3AQP as the first non-enzyme member of the very short list of proteins involved in cell death regulation in trypanosomatids.

## Figures and Tables

**Figure 1:**
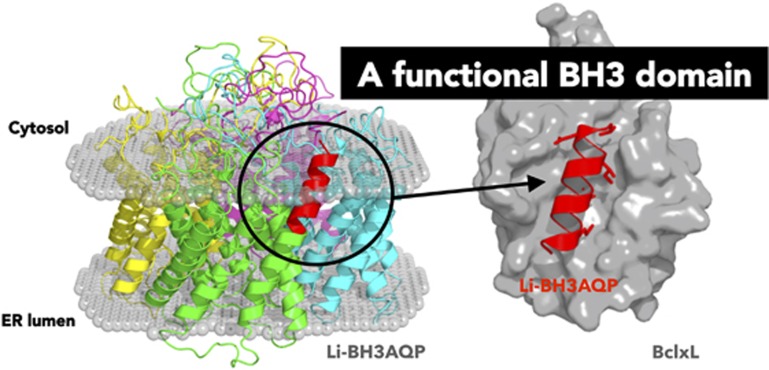
Cartoon representation of a molecular model of a Li-BH3AQP tetramer (left), with each monomer displayed in a different color, inserted into a lipid bilayer for which only the polar heads are depicted as spheres (http://opm.phar.umich.edu/server.php). The BH3 domain from the monomer colored in green is highlighted in red to facilitate comparison with the BH3 domain in complex with Bcl-X_L_ (right). The most relevant residues in the BH3 motif signature are shown as sticks
